# The interrelationships between sleep regularity, obstructive sleep apnea, and hypertension in a middle-aged community population

**DOI:** 10.1093/sleep/zsae001

**Published:** 2024-01-05

**Authors:** Kelly Sansom, Amy Reynolds, Daniel Windred, Andrew Phillips, Satvinder S Dhaliwal, Jennifer Walsh, Kathleen Maddison, Bhajan Singh, Peter Eastwood, Nigel McArdle

**Affiliations:** Centre for Sleep Science, School of Human Sciences, University of Western Australia, Perth, WA, Australia; Queen Elizabeth II Medical Centre, West Australian Sleep Disorders Research Institute, Nedlands, WA, Australia; Flinders University, College of Medicine and Public Health, Flinders Health and Medical Research Institute - Sleep Health, Adelaide, SA, Australia; Flinders University, College of Medicine and Public Health, Flinders Health and Medical Research Institute - Sleep Health, Adelaide, SA, Australia; School of Psychological Sciences, Monash University, Turner Institute for Brain and Mental Health, Clayton, VIC, Australia; School of Psychological Sciences, Monash University, Turner Institute for Brain and Mental Health, Clayton, VIC, Australia; Curtin Health Innovation Research Institute, Faculty of Health Sciences, Curtin University, Bentley, WA, Australia; Office of the Provost, Singapore University of Social Sciences, Clementi, Singapore; Duke-NUS Medical School, National University of Singapore, Singapore; Institute for Research in Molecular Medicine (INFORMM), Universiti Sains Malaysia, Pulau Pinang, Malaysia; Centre for Sleep Science, School of Human Sciences, University of Western Australia, Perth, WA, Australia; Queen Elizabeth II Medical Centre, West Australian Sleep Disorders Research Institute, Nedlands, WA, Australia; Department of Pulmonary Physiology and Sleep Medicine, Sir Charles Gairdner Hospital, Perth, WA, Australia; Centre for Sleep Science, School of Human Sciences, University of Western Australia, Perth, WA, Australia; Queen Elizabeth II Medical Centre, West Australian Sleep Disorders Research Institute, Nedlands, WA, Australia; Department of Pulmonary Physiology and Sleep Medicine, Sir Charles Gairdner Hospital, Perth, WA, Australia; Centre for Sleep Science, School of Human Sciences, University of Western Australia, Perth, WA, Australia; Queen Elizabeth II Medical Centre, West Australian Sleep Disorders Research Institute, Nedlands, WA, Australia; Department of Pulmonary Physiology and Sleep Medicine, Sir Charles Gairdner Hospital, Perth, WA, Australia; Health Futures Institute, Murdoch University, Perth, WA, Australia; Centre for Sleep Science, School of Human Sciences, University of Western Australia, Perth, WA, Australia; Queen Elizabeth II Medical Centre, West Australian Sleep Disorders Research Institute, Nedlands, WA, Australia; Department of Pulmonary Physiology and Sleep Medicine, Sir Charles Gairdner Hospital, Perth, WA, Australia

**Keywords:** sleep regularity, patterns, actigraphy, hypertension, obstructive sleep apnea

## Abstract

**Study Objectives:**

Little is known about the interrelationships between sleep regularity, obstructive sleep apnea (OSA) and important health markers. This study examined whether irregular sleep is associated with OSA and hypertension, and if this modifies the known association between OSA and hypertension.

**Methods:**

Six hundred and two adults (age mean(SD) = 56.96(5.51) years, female = 60%) from the Raine Study who were not evening or night shift workers were assessed for OSA (in-laboratory polysomnography; apnea–hypopnea index ≥ 15 events/hour), hypertension (doctor diagnosed, or systolic blood pressure ≥140 mmHg and/or diastolic ≥90 mmHg) and sleep (wrist actigraphy for ≥5 days). A sleep regularity index (SRI) was determined from actigraphy. Participants were categorized by tertiles as severely irregular, mildly irregular, or regular sleepers. Logistic regression models examined the interrelationships between SRI, OSA and hypertension. Covariates included age, sex, body mass index, actigraphy sleep duration, insomnia, depression, activity, alcohol, smoking, and antihypertensive medication.

**Results:**

Compared to regular sleepers, participants with mildly irregular (OR 1.97, 95% confidence intervals [CI] 1.20 to 3.27) and severely irregular (OR 2.06, 95% CI: 1.25 to 3.42) sleep had greater odds of OSA. Compared to those with no OSA and regular sleep, OSA and severely irregular sleep combined had the highest odds of hypertension (OR 2.34 95% CI: 1.07 to 5.12; *p* for interaction = 0.02) while those with OSA and regular/mildly irregular sleep were not at increased risk (*p* for interaction = 0.20).

**Conclusions:**

Sleep irregularity may be an important modifiable target for hypertension among those with OSA.

## Graphical Abstract

**Graphical Abstract:**
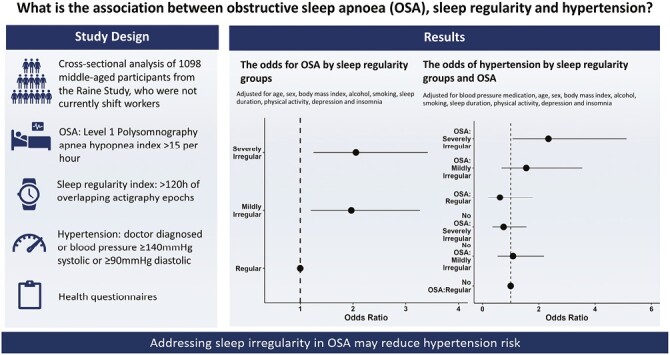

**Statement of Significance**

Sleep regularity has emerged as an important independent dimension of sleep health. Studies have associated sleep irregularity with increased risk of hypertension and cardiovascular disease. However, no study to date has explored the association between sleep regularity, assessed over a 24-hour period, and OSA and whether this modifies the known association between OSA and hypertension. In the present study we show that sleep irregularity and OSA commonly co-occur and compared to individuals with regular sleep and no OSA those who had severely irregular sleep and OSA had greater odds of hypertension while those with OSA and regularly/mildly irregular sleep did not have increased risk of hypertension. Severely irregular sleep may be an important consequence of OSA-related sleep disruption and may contribute to increased risk of hypertension.

## Introduction

Obstructive sleep apnea (OSA) is a prevalent sleep-breathing disorder estimated to impact one billion middle-aged adults globally [1]. It is characterized by intermittent episodes of partial or complete upper airway obstruction resulting in hypoxemia, large negative intrathoracic pressure swings, and sleep fragmentation. The secondary effects of OSA include overactivation of the sympathetic nervous system, surges in blood pressure (BP), oxidative damage, and inflammation. These injurious physiological consequences contribute to the development of hypertension and significant cardiovascular sequelae including myocardial infarction and stroke [2]. However, not all patients with OSA present with the same risk of hypertension [3–7] and not all experience a reduction in BP [8] after using the gold-standard treatment of continuous positive airway pressure. Therefore, a better understanding of additional sleep-related factors associated with OSA-related hypertension may facilitate precision medicine approaches.

Studies in shift workers [9] and the general community [10–14] have identified associations of sleep irregularity with hypertension and increased risk of adverse cardiovascular outcomes. It is thought that individuals with higher day-to-day variability in sleep and wake times (“sleep irregularity”) may experience misalignment between their endogenous circadian rhythm and other behaviors including sleep, eating and physical activity, which could contribute to hypertension [15, 16]. These behavioral changes associated with irregular sleep may also result in weight gain which could predispose individuals to OSA. Alternatively, OSA may contribute to sleep irregularity as a major consequence of OSA is impaired cognitive functioning and judgment which may lead to poor decision-making, and behaviors [17]. Also, key symptoms related to OSA [18] including daytime sleepiness and comorbid insomnia may increase random daytime napping or frequent waking during the night leading to irregular sleep patterns. Furthermore, OSA-related hypoxemia has been associated with dysregulation of circadian clock proteins which might also contribute to irregular sleep timing [19–22]. Therefore, sleep irregularity may be associated with OSA and/or might contribute to increased OSA severity and risk of poor health outcomes such as hypertension.

A recently developed sleep metric, the sleep regularity index (SRI) can be derived from actigraphy and provides the opportunity to assess sleep regularity in large cohort studies [23, 24]. The SRI calculates the percentage probability of an individual being awake or asleep at any two time points 24 hours apart [23]. SRI scores typically range from 0 to 100, with higher values reflecting greater regularity. The advantage of using this objective metric is that it captures day-to-day changes in sleep patterns and does not make assumptions about how many sleep episodes a person has over a 24-hour period [25]. This makes the SRI well-suited for populations with fragmented sleep, such as those with OSA [24]. We are not aware of any study to date that has examined the association between the SRI and OSA with hypertension.

The primary aim of the present study was to examine the independent associations between sleep regularity with both OSA and hypertension in a community sample without extreme forms of circadian misalignment from shift work. The secondary aims were to determine the combined association between sleep irregularity and OSA with prevalent hypertension and if the association between OSA and hypertension is modified by sleep regularity. We hypothesized that OSA and hypertension would be independently associated with sleep irregularity and that sleep irregularity may modify the association between OSA and prevalent hypertension.

## Materials and Methods

### Study design and participants

Participants were from the Raine Study, a multigeneration prospective cohort study of parents (generation 1, or gen1) and their children (generation 2, or gen2). Details of the study are available elsewhere [26]. Briefly, gen1 mothers were recruited between 1989 and 1992 in Perth, Western Australia, and gave birth to the offspring cohort (gen2). Follow-up data were collected on gen1 mothers and fathers (*n* = 1098) between 2014 and 2017 at a time when their gen2 children were 26 years of age (gen1-26 year follow-up). The gen1-26-year follow-up included 1 night of level one polysomnography (PSG), BP measurements, 7 days of actigraphic monitoring, and detailed health questionnaires. This research was approved by the University of Westerns Australia Human Research Ethics Committee on April 29, 2020 and provides a single consolidated approval (RA/4/20/5722) for use of research data and/or biosamples held in the Raine Study data collection. Participants were excluded from the primary analysis if they self-reported evening or night shift work or were missing data required for analysis.

### Study evaluations

#### Level one polysomnography (PSG).

Polysomnography (PSG) was performed using the Compumedics Grael sleep monitoring system (Grael Compumedics, Abbotsford, Victoria, Australia) at the Center for Sleep Science, The University of Western Australia. Participants turned their lights out at their preferred time, but before midnight, and were woken at 06:00 hours or later. Experienced sleep scientists scored the PSG studies according to the 2012 American Academy of Medicine recommended sleep guidelines [27]. OSA was defined as an apnea–hypopnea index (AHI) of ≥15 events/hour. Time spent with oxygen saturation under 90% (T90, minutes) and the respiratory arousal index (events/hour) were used as severity markers of OSA (scores were dichotomized at the median).

#### BP and hypertension evaluation.

BP was assessed in the afternoon before the PSG (14:30–18:00 hours), in the evening before sleep (21:00–23:00 hours) and in the morning after the PSG (05:30–7:00 hours) using validated equipment and aligned with recommended BP measurement techniques [28]. At each measurement timepoint, the first BP measure was discarded, and the remaining measures were averaged to calculate systolic and diastolic BP. The average systolic and diastolic BP values from the afternoon, evening, and morning were then averaged to derive overall systolic and diastolic BP. “Doctor diagnosed” hypertension was obtained from a medical questionnaire in which participants were asked “has a doctor diagnosed you with high blood pressure?” Prevalent hypertension was defined as (1) an overall elevated systolic (≥140 mmHg) or diastolic (≥90 mmHg) BP [28]; and/or (2) responding “yes” to “doctor diagnosed” hypertension.

#### Actigraphy.

Sleep and wake patterns were obtained from wrist actigraphy (GT3X + ActiGraph LLC, Pensacola, FL; sampling frequency 30 Hz; idle sleep mode not enabled) and were collected on the non-dominant wrist during the PSG night and for the subsequent seven nights in the home setting. Participants were instructed to complete a self-reported sleep diary of lights off and wake time each day. After data collection, the ActiGraph data were downloaded into the manufacturer’s software ActiLife (ActiGraph LLC, Pensacola, FL; version 6.13.4) and exported as GT3X and CSV file formats.

#### Sleep–wake data.

Sleep–wake data required for calculating the SRI were obtained from the actigraphy raw data CSV files using the open-source package GGIR (version 2. 0.0) [29, 30] in R studio (RStudio Team 2018, Boston MA, R version 4.0.4; code details provided in supplement). The GGIR heuristic algorithm (HDCZA, see van Hees et al. [31] for further details) was used to guide the actigraphy-derived sleep and wake times, as per a previous publication [25] which used this method to derive sleep–wake times for SRI calculation. Individuals with non-wear time of >16 hours from noon to noon (cleaning code two in GGIR), a sleep window <2 hours or >13 hours, or sleep duration of <1 hour or >12 hours were excluded [32].

#### The sleep regularity index.

**Figure 1.**
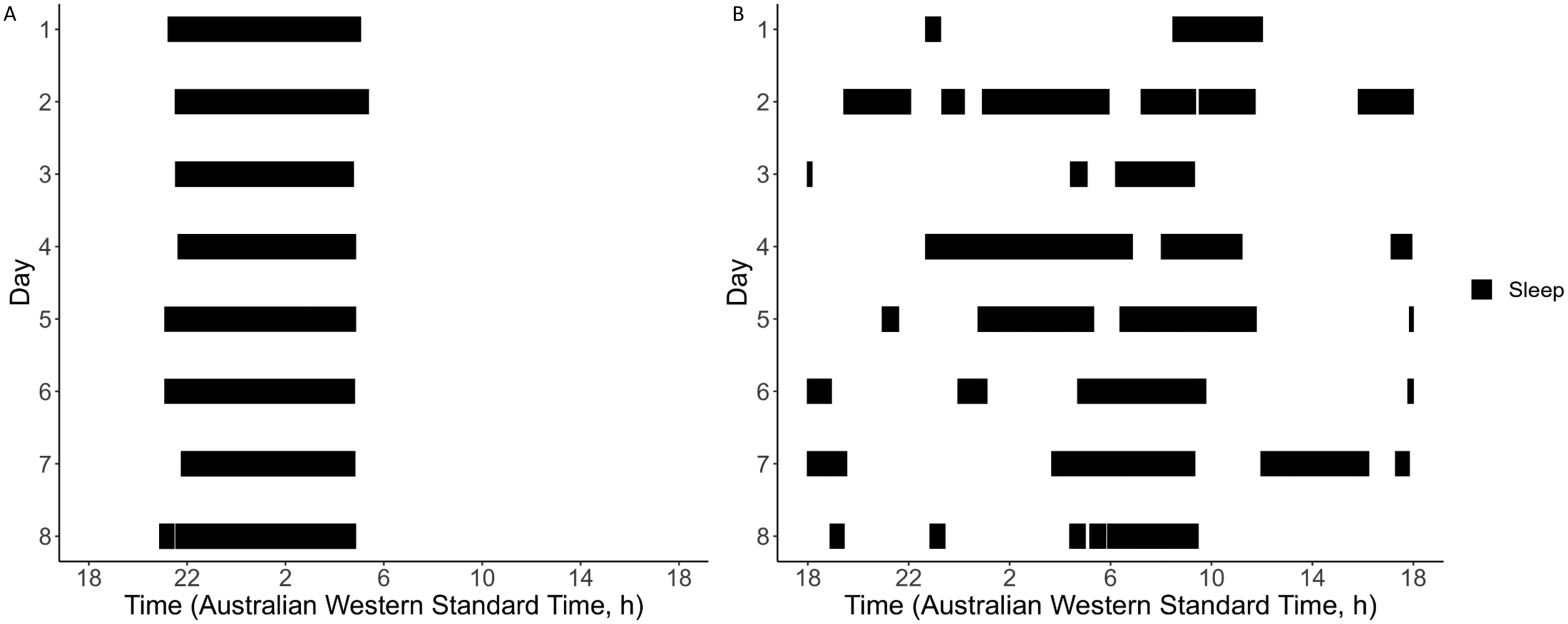
Raster plot of an irregular and regular sleep pattern. Panel A represents a regular sleeper with an SRI score of 94.8 and panel B represents an individual with a sleep regularity index (SRI) score of 24.5 (irregular). The solid lines represent episodes of sleep.

The SRI was determined using the open-source package *sleepreg* [25] (version 1.3.5) in R studio (code available in supplement). *Sleepreg* calculates the SRI based on sleep–wake epoch data from the GGIR analysis output. In contrast to GGIR, the *sleepreg* package allows for analysis of more than one sleep episode per 24-hour period and thus can incorporate naps, fragmented sleep, and/ or awakenings. The SRI was determined using a previously published method [23]. The SRI score ranges from −100 to 100, with lower scores reflecting sleep irregularity and 100 indicating perfectly regular sleep. Example raster plots in Figure 1 of two participants, from the present study, show the contrast of sleep and wake times for regular and irregular sleep patterns over a week. Individuals with <5 days (120 hours of overlapping valid epochs) of sleep–wake data were excluded from the analysis [25]. For ease of interpretation, we categorized the SRI score by tertiles whereby the lowest tertile included participants with severely irregular sleep, the middle tertile included those with mildly irregular sleep and the highest tertile included those with the most regular sleep.

#### Sociodemographic and health information.

Questionnaires completed ≤1 month prior to the PSG were used to obtain sociodemographic and health data. This included information on ethnicity (Caucasian, Aboriginal, Polynesian, Vietnamese, Chinese, Indian, other), age, sex, annual personal income (Australian Dollars; low, <$31 999; medium, $31 200–$64 999; High, >$65 000) and education level (high school or less, training after school, and university). Current smoking was assessed by asking “are you a current smoker?” and participants were categorized as current or non-current smokers (i.e. never or past smokers).

Daily alcohol consumption was obtained from the Australian version of the food frequency questionnaire (Dietary Questionnaire for Epidemiological Studies [33] and participants were categorized as abstainers (<1 drink/day), moderate consumers (≥1 and ≤ 4 drinks/day) or high consumers (>4 standard drinks/day). The International Physical Activity Questionnaire short form was used to categorize participants as having low, moderate, or high physical activity levels [34].

Participants who reported “yes” to doing shift work were classified as shift workers. Those who responded yes to shift work were further classified as day (occurs any time between 6 am and 7 pm), evening (occurs any time between 3 pm and midnight) or night (any 8–10-hour shift between 10 pm and 8 am or any 12-hour shift between 7 pm and 9 am) shift workers. Participants could report more than one type of shift. For the primary analysis, individuals who reported evening and night shifts were excluded as the primary aim was to capture the effect of mild forms of circadian misalignment in the community rather than circadian disruption resulting from shift work. The presence of insomnia symptoms and depression symptoms were based on the Insomnia Symptom Questionnaire [35] and the Depression Anxiety Stress Scale -21 (DASS-21) [36], respectively. BMI was calculated from height and weight measurements collected by research assistants immediately prior to the PSG.

### Statistical analysis

Statistical analysis was performed in Rstudio (Rstudio Team 2018, Boston MA) with R version 4.0.4 (R Core Teams 2021, Vienna Austria). Sociodemographic and sleep characteristics are presented as mean (SD) for continuous variables and count (%) for categorical variables. Comparisons were assessed using a one-way ANOVA for continuous variables and Pearson’s Chi-squared test for categorical variables. Pairwise comparisons were performed for variables with significant differences between sleep regularity groups using unpaired *t*-tests and Chi-squared tests for categorical variables.

Logistic regression was performed to examine the associations between sleep regularity groups, OSA (AHI ≥ 15 events/hour), markers of OSA severity (respiratory arousal index ≥ median; T90 ≥ median) and prevalent hypertension. Multiplicative interaction was examined between the sleep regularity groups and binary OSA status, using a threshold of ≥ 15 events/hour. Interactions were also examined using the SRI and AHI as continuous variables. Furthermore, we also performed a joint analysis between sleep regularity groups and OSA on risk of hypertension with regular sleepers and no OSA as the reference group.

Logistic regression models were adjusted for covariates associated with OSA and/or hypertension including, age, sex, BMI, alcohol consumption, depressive symptoms, insomnia symptoms, self-reported physical activity, actigraphy-derived sleep duration, and smoking status. Models with hypertension as an outcome were additionally adjusted for prescribed BP medication and adjusted for AHI in the model between sleep regularity and hypertension. Variables were excluded if multicollinearity existed [37] (variance inflation factors > 10) and the remaining variables were selected with backward elimination (R packages *olsrr* and *blorr*) [38] using a threshold *p*-value of < 0.05 for all models [39]. The logistic regression results were presented as odds ratios (OR), 95% confidence intervals (CI) and *p*-values.

## Results

### Study participants

**Figure 2.**
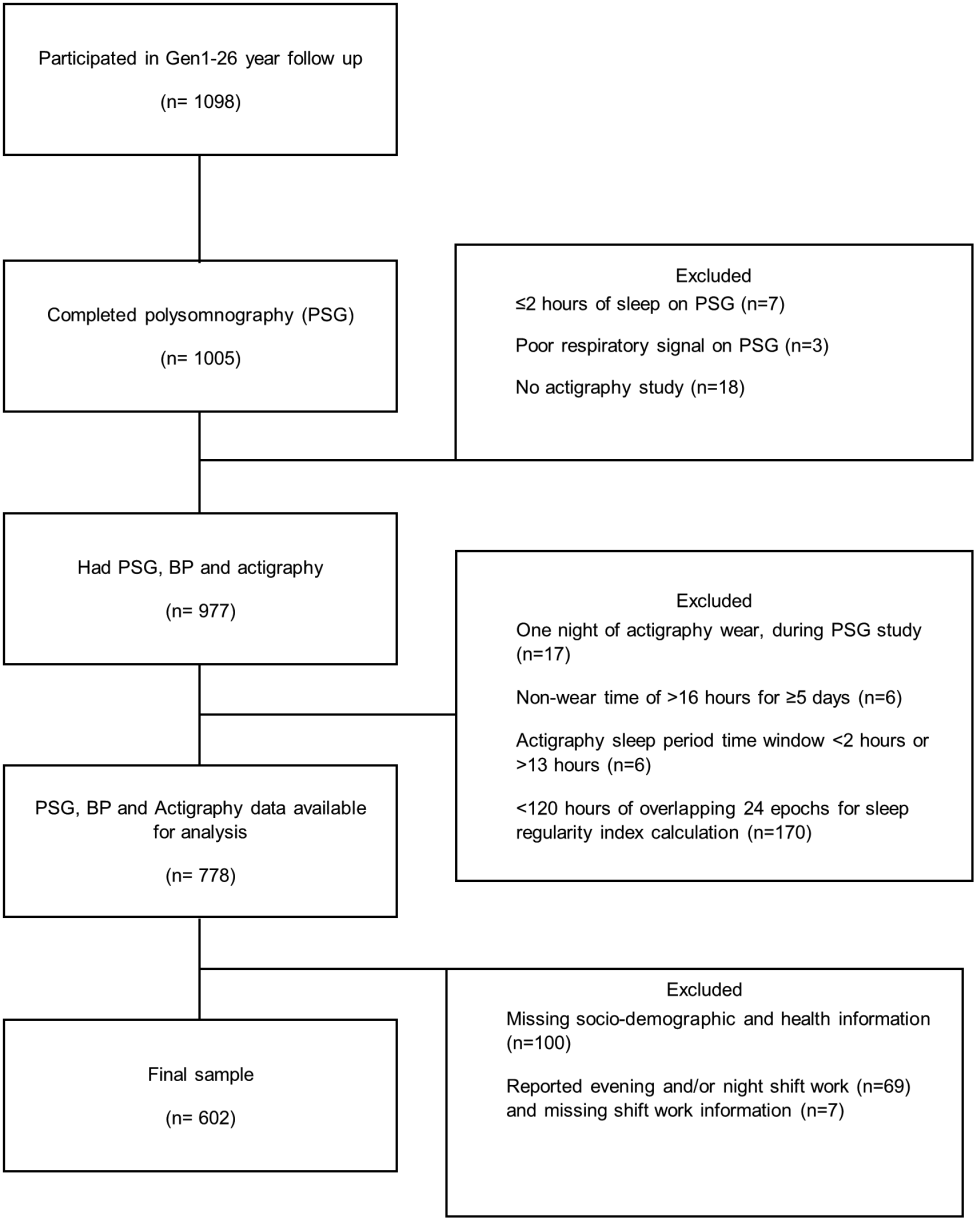
Participant flow diagram.

Participants and study flow (Figure 2): 1098 gen1 parents participated in the gen1-26-year follow-up, of whom 778 had level one PSG, BP and actigraphy (≥120 hours of overlapping epochs) data available for diagnosis of OSA, hypertension and calculation of the SRI, respectively. Of those 778 participants, 100 participants were excluded due to missing sociodemographic and health information required for fully adjusted logistic regression models. A further 76 participants were excluded due to reporting evening or night shift work (*n* = 69) or missing shift work (*n* = 7) data (shift work summarized in Supplementary Table S1). The 602 participants included in the study were similar to those excluded except for having higher education levels and a lower proportion of current smokers (Supplementary Table S2).

### Sociodemographic and health characteristics

The mean (SD) age of participants was 56.96 (5.51) years old, 60% were females, and 92% were Caucasian. Table 1 summarizes the demographic and health characteristics of the study sample stratified by sleep regularity groups. Participants with mildly irregular (*p* = 0.042) and severely irregular (*p* < 0.001) sleep had higher BMI compared to those with regular sleep. In contrast to regular and mildly irregular sleep, those with severely irregular sleep had lower levels of education attainment (*p* = 0.026, *p* = 0.031, respectively), lower income (*p* < 0.001, *p* = 0.006, respectively), higher prevalence of current smoking (*p* = 0.017, *p* = 0.003, respectively) and greater self-reported depression symptoms (*p* = 0.007, *p* = 0.003, respectively).

**Table 1.** Sociodemographic and Health Characteristics of Final Sample

	Sleep regularity groups			
Characteristic	Regular, *N* = 200^1^	Mildly irregular, *N* = 201^1^	Severely irregular, *N* = 201^1^	*P*-value^2^
Sex [female]	122 (61%)	114 (57%)	123 (61%)	0.6
Age, years	57.2 (5.2)	57.1 (5.5)	56.6 (5.8)	0.4
BMI, kg/m^2^	26.7 (4.8)	28.3 (5.6)	29.5 (6.2)	<0.001
Ethnicity				0.2
Caucasian	190 (95%)	184 (92%)	178 (89%)	
Aboriginal	1 (0.5%)	0 (0%)	1 (0.5%)	
Polynesian	2 (1.0%)	0 (0%)	1 (0.5%)	
Vietnamese	0 (0%)	1 (0.5%)	0 (0%)	
Chinese	3 (1.5%)	7 (3.5%)	8 (4.0%)	
Indian	3 (1.5%)	8 (4.0%)	12 (6.0%)	
Other	0 (0%)	1 (0.5%)	0 (0%)	
* Unknown*	1	0	1	
Education				0.013
* High school or less*	34 (17%)	42 (21%)	57 (28%)	
* Training after school*	80 (40%)	59 (30%)	72 (36%)	
* University*	85 (43%)	96 (49%)	72 (36%)	
* Unknown*	1	4	0	
Income				<0.001
* Low [<$31 999, AUD]*	37 (19%)	54 (28%)	83 (42%)	
* Middle [$31 200–$64 999, AUD]*	70 (36%)	47 (24%)	48 (24%)	
* High [>$65 000, AUD]*	86 (45%)	95 (48%)	69 (34%)	
* Unknown*	7	5	1	
Smoker [current]	12 (6.0%)	13 (6.5%)	27 (13%)	0.012
Alcohol consumption				0.065
* Low [<1 drink/day]*	68 (34%)	67 (33%)	66 (33%)	
* Moderate [≥1 and ≤ 4 drinks/day]*	105 (52%)	108 (54%)	90 (45%)	
* High [>4 standard drinks/day]*	27 (14%)	26 (13%)	45 (22%)	
Activity (IPAQ)^*^				0.2
* Low*	55 (28%)	53 (26%)	57 (28%)	
* Moderate*	75 (38%)	79 (39%)	59 (29%)	
* High*	70 (35%)	69 (34%)	85 (42%)	
Hypertension	46 (23%)	66 (33%)	64 (32%)	0.058
Depression (DASS score)	3.8 (5.4)	3.7 (5.3)	5.4 (7.0)	0.006

^1^ *n* (%); mean (SD).

^2^Pearson’s Chi-squared test; one-way ANOVA.

BMI, body mass index; AUD, Australian Dollar; IPAQ, International Physical Activity Questionnaire; DASS, depression, anxiety, and stress scale.

^*^Activity categories were defined according to IPAQ short form guidelines [34].

### Sleep characteristics

The sleep-related characteristics of participants in each sleep regularity group are presented in Table 2. The SRI scores ranged from 79.98 to 94.8 for regular, 71.12 to 79.98 for mildly irregular, and 9.45 to 71.07 for severely irregular sleep patterns. Compared to regular sleep and mildly irregular sleep, severely irregular sleep was associated with decreased actigraphy-derived sleep duration (*p* < 0.001, *p* = 0.025, respectively) and poorer self-reported sleep quality (*p* = 0.002, *p* = 0.019, respectively). Relative to those with regular sleep, individuals with mildly irregular and severely irregular sleep had a greater AHI, (*p* ≤ 0.001, *p* < 0.001, respectively), greater T90 (*p* = 0.002, *p* ≤ 0.001, respectively), decreased PSG total sleep time (*p* = 0.009, *p* = 0.030, respectively) and reduced REM sleep (*p* = 0.022, *p* = 0.002, respectively). Those categorized as having severely irregular sleep had increased NREM sleep stage one (*p* = 0.010) and a greater portion of participants with an evening chronotype (*p* < 0.001) when compared to those with regular sleep.

**Table 2.** Sleep-Related Characteristics of Final Sample

	Sleep Regularity Groups			
Variable	Regular, *N* = 200	Mildly irregular, *N* = 201^1^	Severely irregular, *N* = 201^1^	*P*-value^2^
Sleep regularity index	84.8 (3.4)	76.1 (2.6)	61.9 (8.9)	<0.001
PSG total sleep time (hours)	6.2 (0.9)	5.9 (0.9)	5.9 (1.0)	0.008
Actigraphy sleep duration (hours)	7.1 (0.7)	7.0 (0.8)	6.6 (1.3)	<0.001
AHI (events/hour)	10.0 (10.6)	15.5 (17.1)	16.4 (18.1)	<0.001
Time Spo2 < 90% saturation (min)	1.0 (3.4)	4.6 (16.5)	9.6 (38.2)	0.002
Sleep efficiency (%)	81.7 (17.4)	80.1 (21.5)	78.4 (14.5)	0.2
Sleep onset latency	69.4 (40.1)	78.1 (44.1)	73.7 (44.0)	0.13
Wake after sleep onset (min)	15.7 (16.4)	17.7 (18.1)	16.8 (18.6)	0.5
NREM (%)	80.3 (5.6)	81.9 (6.7)	82.3 (6.4)	0.003
N1 (%)	15.4 (7.6)	18.7 (12.4)	18.5 (10.8)	0.003
N2 (%)	50.3 (7.8)	48.4 (9.6)	49.5 (9.8)	0.10
N3 (%)	14.5 (8.0)	14.9 (9.4)	14.3 (9.2)	0.8
REM (%)	19.7 (5.6)	18.1 (6.7)	17.7 (6.4)	0.003
Insomnia symptoms (ISQ)	24 (12%)	29 (14%)	36 (18%)	0.2
Chronotype (MEQ)				0.002
*Morning*	90 (45%)	84 (42%)	70 (35%)	
*Evening*	18 (9.0%)	31 (16%)	47 (24%)	
*Intermediate*	92 (46%)	85 (42%)	81 (41%)	
*Unknown*	0	1	3	
Sleepiness (ESS)	15 (7.5%)	20 (10%)	17 (8.5%)	0.7
*Unknown*	0	1	1	
Poor self-reported sleep quality (PSQI)	70 (35%)	76 (39%)	100 (51%)	0.003
*Unknown*	1	6	6	

^1^Mean (SD).

^2^One-way ANOVA.

PSG, polysomnography; AHI, apnea hypopnea index; Sp02, oxygen saturation; T90, Time; SpO2, 90% saturation; NREM, non-rapid eye movement; N1, NREM stage 1; N2, NREM stage 2; N3, NREM stage 3; REM, rapid eye movement; ISQ, Insomnia Symptom Questionnaire; MEQ, “Morning Eveningness” Questionnaire; ESS, Epworth Sleepiness Scale; PSQI, Pittsburgh Sleep Quality Index.

### Association between sleep regularity and OSA

**Figure 3.**
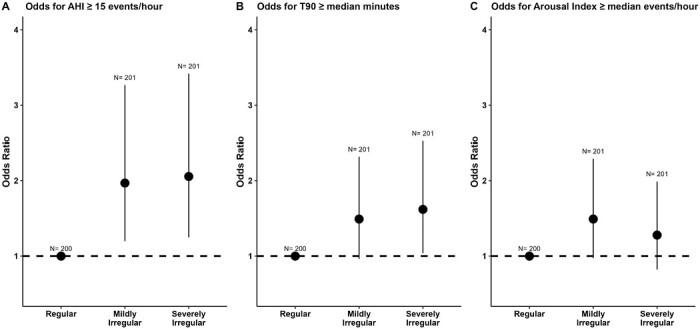
Association between sleep regularity and OSA severity. Odds ratio for obstructive sleep apnea (AHI ≥ 15 events/hour), Time with SpO_2_ < 90% (T90; ≥median) and Arousal index (arousal ≥ median events/hour) across regular = reference group (*n* = 200), mildly irregular (*n* = 201) and severely irregular (*n* = 201) groups. Error bars reflect 95% confidence interval of odds ratio. Variables assessed as potential covariates for models were age, sex, body mass index, alcohol, smoking, physical activity, actigraphy sleep duration, depressive symptoms, and insomnia symptoms. Covariates were excluded if *p*-value > 0.05 or multicollinearity existed.

The odds of OSA were significantly higher in those with mildly irregular sleep (OR 1.97, 95% CI: 1.20 to 3.27, *p* = 0.008) and severely irregular sleep (OR 2.06, 95% CI: 1.25 to 3.42, *p* = 0.0049) relative to those with regular sleep (Figure 3A). When physiological markers of OSA severity were assessed, increased odds for high T90 were identified only in the severely irregular sleep group (OR 1.62 95% CI: 1.04 to 2.53, *p* = 0.033; Figure 3B) and no difference in odds for elevated arousal index across sleep regularity groups (Figure 3C).

### Independent associations between sleep regularity and OSA with hypertension

In the adjusted analysis there was insufficient evidence of an independent association between mildly irregular sleep (OR 1.31, 95% CI: 0.74 to 2.32, *p* = 0.400) and severely irregular sleep (OR 1.17, 95% CI: 0.65 to 2.08, *p* = 0.600) with hypertension relative to those with regular sleep. OSA was independently associated with increased odds of hypertension as a continuous variable (OR 1.4095% CI: 1.05 to 1.87, *p* = 0.022) and there was a trend when OSA was dichotomized at an AHI threshold of 15 events/hour (OR 1.62 95% CI: 0.99 to 2.71, *p* = 0.062).

### Joint effect and interaction of OSA and sleep regularity on hypertension

**Figure 4.**
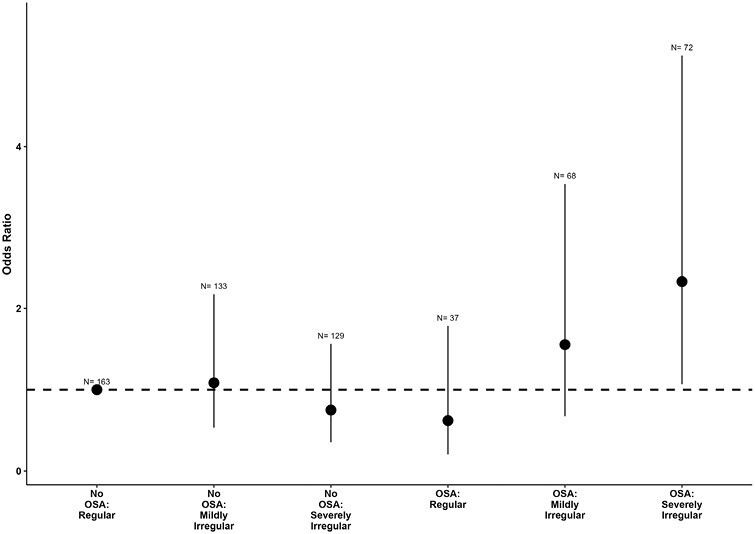
Odds ratio of hypertension across sleep regularity and OSA groups combined. Error bars reflect 95% confidence interval of odds ratio and *N* represents the number of observations per each group. Variables assessed as potential covariates for models were age, sex, body mass index, alcohol, smoking, physical activity, actigraphy sleep duration, depressive symptoms, insomnia symptoms, apnea–hypopnea index, and antihypertensive medication. Covariates were excluded if *p*-value > 0.05 or multicollinearity existed. *N* = number of observations.

There was evidence of a joint effect between OSA and sleep regularity. Compared to those with no OSA and regular sleep, OSA and severely irregular sleep combined had the highest odds of hypertension (OR 2.34 95% CI: 1.07 to 5.12, *p* = 0.033) while those with OSA and regular/mildly irregular sleep were not at increased risk (Figure 4). Furthermore, in a separate model, we included an interaction between OSA and whether a person had mildly irregular or severely irregular sleep on prevalent hypertension. We found a significant interaction between OSA and severely irregular sleep (p for interaction = 0.02) but not for OSA and mildly irregular sleep (*p* interaction = 0.20) on prevalent hypertension (Supplementary Table S3). This interaction was also confirmed in stratified analysis when the relationship between OSA and hypertension was stratified by the three sleep regularity groups (Supplementary Table S4). However, no interaction was found between OSA and sleep regularity when both variables were examined continuously (OR 0.99 95% CI: 0.96 to 1.01, *p* = 0.4).

### Sensitivity analysis

When evening and night shift workers were included (*n* = 69) the independent associations between the sleep regularity groups with OSA and hypertension remained unchanged. However, when assessing the association between OSA severity and sleep regularity groups we found participants with severely irregular sleep no longer had significantly higher odds of T90 ≥ median (OR 1.47, 95% CI: 0.97 to 2.23, *p* = 0.071). Similarly, in the joint analysis, those with OSA and severely irregular sleep combined had the highest odds of hypertension relative to participants with no OSA and regular sleep (OR 2.15, 95% CI: 1.01 to 4.56, *p* = 0.047). A significant interaction between severe sleep irregularity and OSA was also identified (*p* = 0.012). No significant interaction was identified when continuous variables for SRI and AHI were used (OR 0.99, 95% CI: 0.97 to 1.01, *p* = 0.30).

## Discussion

To the best of our knowledge, this is the first report of the associations between the SRI and OSA and their associations with hypertension in a large, well-characterized, middle-aged community population. Participants have previously been shown to be representative of the Australian population with the exception of slightly higher socioeconomic status [26, 40]. The main study findings were (1) there is an association between OSA and irregular sleep patterns and (2) participants with both OSA and severely irregular sleep patterns had significantly higher odds of prevalent hypertension relative to those with regular sleep patterns and no OSA. We also found a statistically significant interaction between severely irregular sleep and OSA on prevalent hypertension. These findings suggest that sleep regularity, as assessed by the SRI, may be an important modifier of the known association between OSA and hypertension.

The SRI scores observed in the present study (mean (SD), 74.0 [11.0]) are comparable with scores from other large predominantly middle-aged community-based populations [12, 25]. Consistent with other cohorts, the distribution of SRI scores was negatively skewed, indicating that individuals with extremely irregular sleep were much less frequent than those with regular sleep patterns. As in the present study, participants from the UK Biobank study [25] with lower SRI scores had a lower reported income and educational attainment relative to those with regular sleep schedules. Additionally, in the present study, we found a higher proportion of smoking, alcohol consumption, evening chronotype, and poor self-reported sleep among participants with severely irregular sleep. These findings suggest that decreased sleep regularity may be associated with socioeconomic disadvantage and behaviors associated with negative health outcomes.

The current study shows for the first time that both mildly irregular sleep and severely irregular sleep are associated with approximately double the odds of OSA relative to regular sleep. Irregular sleep patterns may contribute to circadian disruption and lead to changes in sleep composition and quality [23]. Such changes may impact the amount of rapid eye movement and non-rapid eye movement, which may in turn predispose to abnormal control of breathing [41]. In a study of college-aged men, those with greater variability in sleep timing according to retrospective questionnaire had significantly less slow-wave sleep than regular sleepers [41]. It is well-recognized that breathing is more stable during slow-wave sleep and fewer obstructive respiratory events occur relative to other stages of sleep [42, 43]. However, we did not identify differences in the proportions of slow-wave sleep between the regular and irregular sleepers in the present study, this may relate to the recording of sleep in a laboratory environment thus this could be investigated in future studies conducted in the home environment.

Alternatively, it is biologically plausible that OSA could predispose individuals to irregular sleep. OSA is known to impair sleep quality, cognitive function, and judgment [17], which could lead to behavioral changes such as irregular sleep timing. Furthermore, common symptoms associated with OSA [18] including daytime sleepiness and comorbid insomnia may alter sleep and wake patterns over a 24-hour period leading to irregular sleep patterns. OSA may also disrupt circadian rhythms, which in turn may contribute to irregular sleep schedules. Indeed, there is preliminary evidence of overexpression of circadian clock gene proteins in patients with OSA, relative to controls [44], perhaps secondary to the effects of intermittent hypoxia [19–22]. Furthermore, while the SRI is a composite measure of sleep regularity, which is sensitive to sleep fragmentation, notably there was very little relationship between WASO and SRI in our study. Therefore, the observed relationship between OSA and SRI may not simply be explained by sleep fragmentation caused by OSA. Further prospective studies are required to delineate whether sleep regularity is a consequence of OSA or if there is a bidirectional relationship.

Our study did not demonstrate an independent association between the SRI and odds of hypertension in adjusted analyses. In contrast, Lunsford-Avery et al [12] reported greater rates of hypertension and cardiovascular disease among MESA study participants with lower SRI scores after adjustment for age, sex, and ethnicity. A number of studies using other measures of sleep regularity, such as standard deviation of sleep duration [11, 14, 45] and interdaily stability [10, 13], also reported higher BP and elevated odds of hypertension, respectively, in those with poor sleep regularity. The inconsistency in findings may relate to methodological differences. Firstly, the MESA study participants used in the study by Lunsford-Avery et al. [12] were older than participants in the present study. Secondly, Lunsford-Avery et al. [12] did not account for OSA which may be an important confounder as we demonstrate OSA is associated with sleep regularity and OSA is a known risk factor for hypertension [46]. Thirdly, studies that used other measures of sleep regularity [10, 11, 13, 14], not the SRI, may capture different components of sleep regularity and hence result in different associations between sleep regularity and hypertension [24].

Given OSA can disrupt sleep, and previous studies have associated irregular sleep with hypertension [10–14], we investigated whether SRI and OSA combine to increase the risk of prevalent hypertension. We found that compared to those with regular sleep and no OSA, participants with severely irregular sleep patterns and OSA had the greatest odds of hypertension while those with regular/mildly irregular sleep patterns and OSA were not at elevated risk of prevalent hypertension. We also observed a significant interaction between severely irregular sleep and OSA on prevalent hypertension. Importantly, those with no OSA and irregular sleep did not have increased odds of hypertension. Therefore, these findings suggest that sleep irregularity may be an important consequence of OSA-related sleep disruption and may increase the risk of hypertension. However, given the cross-sectional nature of the present study, there is a need for prospective evidence to determine the direction of these associations.

The mechanisms underlying the increased risk of hypertension among individuals with irregular sleep and OSA are unclear. Studies have shown those with irregular sleep, particularly shift workers, experience circadian misalignment which may predispose such individuals to increased risk of hypertension and cardiovascular events [11, 47]. Intervention studies also show that healthy groups who undergo circadian misalignment protocols develop increases in BP [48, 49], in part due to dysregulation of autonomic control. One of the mechanisms whereby OSA can lead to hypertension is through autonomic imbalance [2]. Hence, we speculate that those with co-occurring irregular sleep and OSA may be particularly at risk because of synergistic autonomic disruption. However, it is important to note that causality cannot be determined in the current study design so it may be possible that hypertension contributes to irregular sleep which could increase the risk of OSA. There is evidence of a bidirectional association between OSA and hypertension [50] so further prospective studies are needed to delineate these associations.

A strength of this study is the use of the SRI to quantify variability in sleep timing. The SRI is advantageous relative to other metrics of sleep variability as it can capture day-to-day changes in sleep on a 24-hour timescale. Furthermore, it does not assume one main nocturnal sleep episode but rather accounts for all episodes of sleep throughout a 24-hour period, thus making is suitable for individuals with atypical sleep patterns [25]. Another strength of this study is that OSA was diagnosed using the gold-standard level one PSG, hypertension was assessed based on doctor diagnosis and multiple BP measurements at consistent times, and validated health and lifestyle questionnaires were used. We excluded evening and night shift workers from the primary analysis due to a known elevated risk for hypertension [51, 52] and thus our findings are generalizable to a community sample without extreme circadian disruption. Furthermore, the SRI was calculated using open-source package *sleepreg* and GGIR, which allows for data transparency and reproducible testing by other researchers.

The present study has a number of limitations. Future longitudinal and interventional studies will be needed to determine if there are causal associations between the SRI with OSA and hypertension and provide insight into possible underlying mechanisms underpinning these associations. Moreover, the SRI is a sensitive but nonspecific measure of sleep regularity, meaning one score may capture a range of phenotypes. For example, two individuals with a similar SRI score may be different in that one individual has highly fragmented sleep but consistent sleep and wake times and the other has non-fragmented sleep but inconsistent sleep and wake times each day [24]. As such, future studies should additionally evaluate and compare how other metrics of sleep regularity, such as standard deviation of mid-sleep time, are differentially associated with OSA and hypertension. The present study was limited in ability to calculate other commonly used metrics such as the standard deviation in sleep timing as this requires more than seven nights of actigraphy to provide unbiased estimates of sleep regularity [24]. Also, the SRI was calculated over a minimum of five overlapping nights which means some individuals may not have had weekend and weekday overlap which may possibly result in a less accurate measure of sleep regularity. Furthermore, the sample size of sleep regularity groups was smaller in stratified analyses, which likely explains the large confidence intervals and the potential for type 2 errors. Future studies with larger subgroups may provide more robust estimates. Lastly, sleep times were derived from actigraphy which has inherent limitations in detecting sleep [53] based on the activity of the wrist and does not accurately stage sleep according to electroencephalogram. However, actigraphy is currently the most practical objective measure available for collecting sleep data over long periods in the home setting.

In summary, this large cross-sectional study of middle-aged community participants found irregular sleep patterns were associated with increased odds of OSA. Furthermore, an interaction between OSA and severely irregular sleep patterns on hypertension was observed suggesting that the SRI may modify the well-established association between OSA and hypertension. Irregular sleep may be an important marker of OSA-related sleep disruption and may be an important modifiable health target. The clinical assessment of sleep regularity in the future may be aided by the rise in popularity of commercial wrist-watches or under-bed pressure sensors capable of tracking sleep timing in the home setting. Future studies are required to confirm these associations in prospective designs, understand the underlying mechanisms and determine whether sleep-behavioral interventions may improve health outcomes.

## Supplementary Material

zsae001_suppl_Supplementary_Material

## Data Sharing

We are willing to share data from this study, according to current Raine study data sharing rules. The Raine Study holds a rich and detailed collection of data gathered over 30 years for the purpose of health and well-being research. The informed consent provided by each participant does not permit individual-level data to be made available in the public domain (i.e. a public data repository). However de-identified analytic data sets are available to all researchers for original research or auditing of published findings. All data access is managed through established Raine Study procedures which require data handlers to agree to a code of conduct, outlined in the Raine Study Researcher Engagement Policy, that includes safeguards to protect the identity of participants. Details of the data access processes, and code of conduct are available on the Raine Study website (www.rainestudy.org.au).
