# Supplementary material for: The interrelationships between sleep regularity, obstructive sleep apnea, and hypertension in a middle-aged community population
Source: Sleep. 2024 Jan 5;47(3):zsae001. doi: 10.1093/sleep/zsae001 (PMC10925954; doi:10.1093/sleep/zsae001)
Supplement: zsae001_suppl_Supplementary_Material [file zsae001_suppl_supplementary_material.docx]

**Supplementary material**

**Title:** The interrelationships between sleep regularity, obstructive sleep apnea and hypertension in a middle-aged community population

Authors: Kelly Sansom^1,2,3^, Amy Reynolds^3^, Daniel Windred^4^, Andrew Phillips^4^, Satvinder S. Dhaliwal^5-8^, Jennifer Walsh^1,2,9^, Kathleen Maddison^1,2,9^, Bhajan Singh^1,2,9^, Peter Eastwood^10^, Nigel McArdle^1,2,9^

1. The University of Western Australia, Centre for Sleep Science, School of Human Sciences, Perth, WA, AU
2. 2. Queen Elizabeth II Medical Centre, West Australian Sleep Disorders Research Institute, Nedlands, WA, AU
3. Flinders University, Flinders Health and Medical Research Institute - Sleep Health, Adelaide, SA, AU
4. Monash University, Turner Institute for Brain and Mental Health, School of Psychological Sciences, Clayton, VIC, AU
5. Curtin Health Innovation Research Institute, Faculty of Health Sciences, Curtin University, Bentley, Western Australia, Australia 6102
6. Office of the Provost, Singapore University of Social Sciences, 463 Clementi Road, Clementi, 599494, Singapore
7. Duke-NUS Medical School, National University of Singapore, Singapore 169857
8. Institute for Research in Molecular Medicine (INFORMM), Universiti Sains Malaysia, 11800 Minden, Pulau Pinang, Malaysia
9. Department of Pulmonary Physiology & Sleep Medicine, Sir Charles Gairdner Hospital, Perth, Western Australia, Australia
10. Health Futures Institute, Murdoch University, Perth, WA, Australia

Corresponding author: Kelly Sansom (email, kelly.sansom@flinders.edu.au)

Address: Level 2A, Adelaide Institute for Sleep Health, 5 Laffer Drive, Bedford Park SA 5042

| **Table S1: study sample including evening and night shift workers** | |
| --- | --- |
| Shift type* | N = 671^1^ |
| Night | 5 (0.7%) |
| Evening | 7 (1.0%) |
| Evening and night | 3 (0.4%) |
| Day and night | 10 (1.5%) |
| Day and evening | 19 (2.8%) |
| Morning, evening, and night | 25 (3.7%) |
| Day | 10 (1.5%) |
| No shift work | 592 (88%) |
| ^1^n (%) | |
| * Shift workers were classified as those who responded yes to shift work and were further classified as day (occurs any time between 6am and 7pm), evening (occurs any time between 3pm and midnight) or night (any 8–10-hour shift between 10pm and 8am or any 12-hour shift between 7pm and 9am) shift workers. Participants could report more than one type of shift. | |

| **Table S2: comparison of included and excluded participants** | | | | |
| --- | --- | --- | --- | --- |
|  | | **Inclusion** | |  |
| **Characteristic** | **Overall, N = 1,098^1^** | **Included, N = 602^1^** | **Excluded, N = 496^1^** | **p-value^2^** |
| Sex [female] | 636 (58%) | 359 (60%) | 277 (56%) | 0.2 |
| BMI, kg/m^^2^ | 28.4 (5.7) | 28.2 (5.7) | 28.8 (5.7) | 0.069 |
| *Unknown* | 57 | 0 | 57 |  |
| Ethnicity |  |  |  | 0.3 |
| Caucasian | 1,011 (92%) | 552 (92%) | 459 (93%) |  |
| Aboriginal | 5 (0.5%) | 2 (0.3%) | 3 (0.6%) |  |
| Polynesian | 6 (0.5%) | 3 (0.5%) | 3 (0.6%) |  |
| Vietnamese | 4 (0.4%) | 1 (0.2%) | 3 (0.6%) |  |
| Chinese | 30 (2.7%) | 18 (3.0%) | 12 (2.4%) |  |
| Indian | 34 (3.1%) | 23 (3.8%) | 11 (2.2%) |  |
| Other | 5 (0.5%) | 1 (0.2%) | 4 (0.8%) |  |
| *Unknown* | 3 | 2 | 1 |  |
| Age, years | 56.7 (5.7) | 57.0 (5.5) | 56.3 (6.0) | 0.050 |
| Income |  |  |  | 0.8 |
| *Low [<$31,999, AUD]* | 297 (29%) | 174 (30%) | 123 (28%) |  |
| *Middle [$31,200-$64,999, AUD]* | 290 (28%) | 165 (28%) | 125 (28%) |  |
| *High [>$65,000, AUD]* | 443 (43%) | 250 (42%) | 193 (44%) |  |
| *Unknown* | 68 | 13 | 55 |  |
| Education |  |  |  | <0.001 |
| *Education: High school or less* | 258 (25%) | 133 (22%) | 125 (28%) |  |
| *Education: Training after school* | 399 (38%) | 211 (35%) | 188 (42%) |  |
| *Education: University* | 390 (37%) | 253 (42%) | 137 (30%) |  |
| *Unknown* | 51 | 5 | 46 |  |
| Activity (IPAQ)* |  |  |  | 0.4 |
| *Low* | 280 (27%) | 165 (27%) | 115 (26%) |  |
| *Moderate* | 356 (34%) | 213 (35%) | 143 (32%) |  |
| *High* | 407 (39%) | 224 (37%) | 183 (41%) |  |
| *Unknown* | 55 | 0 | 55 |  |
| Smoker [current] | 108 (10%) | 52 (8.6%) | 56 (13%) | 0.028 |
| *Unknown* | 60 | 0 | 60 |  |
| Alcohol consumption |  |  |  | 0.8 |
| *Low [<1 drink/day]* | 332 (33%) | 201 (33%) | 131 (32%) |  |
| *Moderate [≥1 and ≤4 drinks/day]* | 514 (51%) | 303 (50%) | 211 (51%) |  |
| *High [>4 standard drinks/day]* | 169 (17%) | 98 (16%) | 71 (17%) |  |
| *Unknown* | 83 | 0 | 83 |  |
| ^1^n (%); mean (SD) | | | | |
| ^2^Pearson's Chi-squared test; Welch Two Sample t-test; Fisher's exact test  BMI = body mass index, AUD = Australian Dollar, IPAQ = International Physical Activity Questionnaire  *Activity categories were defined according to IPAQ short form guidelines.^312^ | | | | |
|  | | | | |

| **Table S3: Interaction between sleep regularity and OSA on prevalent hypertension.** | | | |
| --- | --- | --- | --- |
| **Characteristic** | **OR***^1^* | **95% CI***^1^* | **p-value** |
| Sleep regularity groups |  |  |  |
| *Regular* | — | — |  |
| *Severely Irregular* | 0.75 | 0.35, 1.56 | 0.4 |
| *Mildly Irregular* | 1.08 | 0.54, 2.19 | 0.8 |
| OSA |  |  |  |
| *No OSA* | — | — |  |
| *OSA* | 0.62 | 0.21, 1.78 | 0.4 |
| Sleep regularity groups * OSA |  |  |  |
| *Severely Irregular * OSA* | 5.04 | 1.31, 20.6 | 0.021 |
| *Mildly Irregular * OSA* | 2.31 | 0.60, 9.27 | 0.2 |
| *^1^* OR = Odds Ratio, CI = Confidence Interval, OSA = obstructive sleep apnea, N = number of observations.  Variables assessed as potential covariates for models were age, sex, body mass index, alcohol, smoking, physical activity, actigraphy sleep duration, depressive symptoms, insomnia symptoms, apnoea-hypopnea index, and anti-hypertensive medication. Covariates were excluded using backward selection if p-value >0.05 or multicollinearity existed. | | | |

| **Table S4: Association between OSA and hypertension stratified by sleep regularity groups** | | | | | | | | | | | | |
| --- | --- | --- | --- | --- | --- | --- | --- | --- | --- | --- | --- | --- |
| **Characteristic** | **Regular** | | | | **Mildly irregular** | | | | **Severely Irregular** | | | |
|  | **N** | **OR***^1^* | **95% CI***^1^* | **p-value** | **N** | **OR***^1^* | **95% CI***^1^* | **p-value** | **N** | **OR***^1^* | **95% CI***^1^* | **p-value** |
| No OSA | 163 | — | — |  | 133 | — | — |  | 129 | — | — |  |
| OSA | 37 | 0.54 | 0.15, 1.64 | 0.3 | 68 | 1.48 | 0.60, 3.63 | 0.4 | 72 | 3.40 | 1.40, 8.82 | 0.009 |
| *^1^* OR = Odds Ratio, CI = Confidence Interval, OSA = obstructive sleep apnoea, N = number of observations.  Variables assessed as potential covariates for models were age, sex, body mass index, alcohol, smoking, physical activity, actigraphy sleep duration, depressive symptoms, insomnia symptoms, apnoea-hypopnea index, and anti-hypertensive medication. Covariates were excluded using backward selection if p-value >0.05 or multicollinearity existed. | | | | | | | | | | | | |

|  |
| --- |

### Analysis code for *GGIR* and *sleepreg* package in R studio

library(GGIR)

#input
f0 = 1
f1=c()
mode= c(1,2,3,4,5)
datadir = "file path"
outputdir = "file path"


g.shell.GGIR(
 mode=mode,
 datadir= datadir,
 outputdir = outputdir,
 f0=f0,
 f1=f1,
 overwrite= FALSE,
 do.imp = TRUE,
 idloc=2,

 #=====================
 # Part 2
 #=====================
 strategy = 1,
 hrs.del.start = 0, hrs.del.end = 0,
 maxdur = 9,
 includedaycrit = 16, # minimum of 16 hours a day of recording
 qwindow=c(0,24), # all variables will be calculated over the full 24hrs of day
 mvpathreshold =c(100), # moderate-vig physical activity
 bout.metric = 4,
 excludefirstlast = FALSE,
 includenightcrit = 16,
 #=====================
 # Part 3
 #=====================
 timethreshold=c(5,10),
 anglethreshold=5,
 ignorenonwear= TRUE,

 #=====================
 # Part 4
 #=====================
 def.noc.sleep = 1,
 loglocation= "file path",# this code was removed for the analysis without a diary

relyonguider = FALSE,
 sleeplogidnum = FALSE,
 colid=1,
 coln1=2,
 do.visual = TRUE, #create a visual representation of the overlap between the sleeplog entries and the accelerometer detections
 outliers.only = TRUE, # visualise only for nights with a difference in onset or waking time larger than the variable of argument criterror
 criterror = 4, # minimum number of hours difference between sleep log and accelerometer estimate for the night to be included in the visualisation
 nnights = 8,

 #=====================
 # Part 5
 #=====================
 threshold.lig = c(30), threshold.mod = c(100), threshold.vig = c(400),
 boutcriter = 0.8, boutcriter.in = 0.9, boutcriter.lig = 0.8,
 boutcriter.mvpa = 0.8, boutdur.in = c(1,10,30), boutdur.lig = c(1,10),
 boutdur.mvpa = c(1),
 includedaycrit.part5 = 2/3,
 #=====================
 # Visual report
 #=====================
 timewindow = c("WW"), # Changed to add MM
 viewingwindow=1, # midday is in the centre of plot
 visualreport=TRUE,
 dofirstpage = TRUE,
 do.report=c(2,3,4,5))

library(sleepreg)

SRI_from_GGIR(outputdir = "file path")
